# Occurrence of *Aspergillus fumigatus* azole resistance in soils from Switzerland

**DOI:** 10.1093/mmy/myad110

**Published:** 2023-11-01

**Authors:** Stéphanie Schürch, Katia Gindro, Sylvain Schnee, Pierre-Henri Dubuis, Josep Massana Codina, Matthieu Wilhelm, Arnaud Riat, Frédéric Lamoth, Dominique Sanglard

**Affiliations:** Plant Protection Research Division, Mycology Group, Agroscope, 1260 Nyon, Switzerland; Plant Protection Research Division, Mycology Group, Agroscope, 1260 Nyon, Switzerland; Plant Protection Research Division, Mycology Group, Agroscope, 1260 Nyon, Switzerland; Plant Protection Research Division, Mycology Group, Agroscope, 1260 Nyon, Switzerland; Plant Protection Research Division, Mycology Group, Agroscope, 1260 Nyon, Switzerland; Plant Protection Research Division, Mycology Group, Agroscope, 1260 Nyon, Switzerland; Service of Infectious Diseases and Service of Laboratory Medicine, Geneva University Hospitals and Geneva University, 1205 Geneva, Switzerland; Infectious Diseases Service, Department of Medicine, Lausanne University Hospital and University of Lausanne, 1011 Lausanne, Switzerland; Institute of Microbiology, Lausanne University Hospital and University of Lausanne, 1011 Lausanne, Switzerland; Institute of Microbiology, Lausanne University Hospital and University of Lausanne, 1011 Lausanne, Switzerland

**Keywords:** *Aspergillus*, resistance, azoles, fungicides

## Abstract

*Aspergillus fumigatus* is a fungal species causing diverse diseases in humans. The use of azoles for treatments of *A. fumigatus* diseases has resulted in azole resistance. Azoles are also widely used in the environment for crop protection, which resulted in azole resistance. Resistance is primarily due to mutations in *cyp51A*, which encodes the target protein for azoles. Here we addressed the occurrence of azole resistance in soils from a vast part of Switzerland. We aimed to associate the use of azoles in the environment with the occurrence of azole resistance. We targeted sample sites from different agricultural environments as well as sites with no agricultural practice (natural sites and urban sites). Starting from 327 sites, 113 *A. fumigatus* isolates were recovered (2019–2021), among which 19 were azole-resistant (15 with TR34/L98H and four with TR46/Y121F/T289A resistance mutations in *cyp51A*). Our results show that azole resistance was not associated with a specific agricultural practice. Azoles could be chemically detected in investigated soils, however, their presence was not associated with the occurrence of azole-resistant isolates. Interestingly, genetic markers of resistance to other fungicides were detected but only in azole-resistant isolates, thus reinforcing the notion that *A. fumigatus* cross-resistance to fungicides has an environmental origin.

In conclusion, this study reveals the spreading of azole resistance in *A. fumigatus* from the environment in Switzerland. The proximity of agricultural areas to urban centers may facilitate the transmission of resistant strains to at-risk populations. Thus, vigilant surveillance is required to maintain effective treatment options for aspergillosis.

## Introduction

*Aspergillus* species are filamentous fungi (moulds) that are ubiquitous in the environment. They live as saprophytes, although they occasionally infect living hosts including plants, insects, birds, and mammals. Among *Aspergillus* spp., *Aspergillus fumigatus* Freseniu is the most common and life-threatening airborne opportunistic fungal pathogen in humans.^1^

*Aspergillus fumigatus* produces small asexual spores (conidia) that are readily dispersed in the environment and that are easily inhaled through lung tissues in humans. Major sources of *A. fumigatus* conidia are originating from decaying organic matters (e.g., composting sites).^2^ Inhalation of *A. fumigatus* conidia into the lungs can cause multiple diseases that depend on the immune status of the host. These include invasive pulmonary aspergillosis (IA), chronic pulmonary aspergillosis (CPA), and different forms of hypersensitivity diseases such as allergic asthma and allergic bronchopulmonary aspergillosis (ABPA). Some other organs can be affected, such as the sinus, with possible extension to the brain. *Aspergillus* spp. are responsible for over 300 000 cases of IA annually, and global estimates suggest that over 1.2 million patients have CPA. A total of 4.8 million suffer from ABPA with most infections being caused by *A. fumigatus*.^3,4^ While allergic forms of aspergillosis such as ABPA and *Aspergillus* sinusitis are generally not life-threatening, IA is a life-threatening infection: a large prospective study found that the one-year survival rate for immunosuppressed individuals was 59% among solid organ transplant recipients^5^ and 25% among stem cell transplant recipients.^6^

Current antifungal therapy for aspergillosis involves three main antifungal categories: polyenes, echinocandins, and azoles. Among these, azoles are the drug of choice, since they can be administered orally and are critical for long-term treatment.^7^ Azoles target Cyp51A in *A. fumigatus*, an enzyme involved in ergosterol biosynthesis. Several azoles such as itraconazole, voriconazole, posaconazole, and isavuconazole are used to treat and to prevent *A. fumigatus* infections.^8^ Azoles have been beneficial as they improve patient survival, but resistant strains have emerged.^9^ Several resistance profiles have been described, including resistance to specific azoles or multi-azole resistance. After some sporadic reports, an increase in azole resistance appeared starting from 2007 in the Netherlands and in the UK and then later in other countries in all continents.^10^

Azoles are also used as crop protection agents in agriculture in most European countries, in Asia and also in the American continent, although less extensively than in Europe.^3^ Thus, *A. fumigatus* can be in contact with the same substance class in medicine and in the environment and azole resistance can occur in these circumstances. Indeed, resistance to azoles has been detected in numerous fungal plant pathogens after selection due to agricultural azole treatment.^11,12^ In the case of *A. fumigatus*, azole-resistant isolates were recovered from the environment in the Netherlands as early as 2007.^13^ A mutation in the azole target *cyp51A* (L98H), which was associated with a 34-bp tandem repeat (TR34) in the gene promoter, was identified in these isolates.^13^ Interestingly, the same mutation was recovered from patient samples, strongly suggesting that the *cyp51A* TR34/L98H mutation was acquired from environmental isolates. This mutation results in resistance to all medical azoles (pan-azole resistance). Environmental acquisition of azole resistance in the clinic is supported by several studies, one of which illustrated that between 64% and 71% of patients with IA due to an azole-resistant *A. fumigatus* isolate had never received azoles.^14^ Recent reports also suggest the occurrence of other *cyp51A* mutations from environmental isolates with tandem repeat (TRs) in the promoter (e.g., TR46/Y121F/T289A). However, the TR34/L98H mutation is still the most frequent among recovered azole-resistant isolates.^15^ Nevertheless, non-*cyp51A* azole resistance mechanisms have been documented mainly in clinical but much less in environmental isolates to varying degrees (prevalence rates varying from 15% to 60% depending on geographic region).^16–18^ They impact on sterol biosynthesis (e.g., 3-hydroxy-3-methylglutaryl-coenzyme A [HMG-CoA] reductase Hmg1) as well as the regulation of multidrug transporters.^19^ There are concerns that azole resistance could become a global public health threat since fungal spores can disperse easily by circulating air flows across long distances.^1^

Very few studies have addressed up to now the epidemiology of antifungal resistance in *A. fumigatus* in Switzerland from the environment. Our laboratory undertook between 2001 and 2004 a survey of azole resistance from *A. fumigatus* of environmental origins taken from different sites across Switzerland (n = 150). A single isolate resistant to itraconazole and originating from a composting site was recovered but it was still susceptible to other azoles.^20^ This isolate exhibited *cyp51A* mutations (F46Y, M172V, and E427K).^21^ A study that compiled clinical isolates from 2009 to 2011^22^ comprising 3788 *Aspergillus* isolates from 22 centers from 19 countries and including the University Hospital Center of Lausanne (CHUV) concluded a prevalence of azole resistance of 3.6% (range 0.0%–26.1% among the centers). In this study, none of the 580 isolates collected by the CHUV during this time were resistant. That a single Swiss center was involved could explain the absence of azole-resistant isolates in the sampled cohort. Another prospective study performed in 2015 that undertook the detection of azole resistance in soil samples in a limited area in Switzerland (lemanic area) revealed the existence of azole resistance in *A. fumigatus* in approximately 10% of all tested samples (n = 65).^23^ Moreover, the same study revealed azole resistance in two cystic fibrosis patients from the same region. Notably, azole resistance in these patients exhibited a typical environmental signature (TR34/L98H in c*yp51A*), thus suggesting an environmental acquisition of resistance in these patients. These last results were in sharp contrast with the study performed at the CHUV between 2009 and 2011.^20^ A recent clinical study collected respiratory samples from 365 patients from seven Swiss hospitals revealed a 1.1% rate of azole resistance among *A. fumigatus* isolates.^24^ Three out of four of these azole-resistant *A. fumigatus* isolates contained the environmental mutation TR34/L98H.

In this study, our goal was to obtain a more comprehensive estimation of the occurrence of azole resistance in Switzerland in the environment and to explore the potential link between azole use in the environment and the occurrence of azole resistance. To this end, soils were sampled in the entire country between 2019 and 2021. Sites without any recorded human activity as well as sites with different types of agricultural crops with or without azole applications were chosen. Our results showed that azole resistance in *A. fumigatus* could be detected as expected throughout the territory with the occurrence of *cyp51A* mutations typically linked to environmental resistance acquisition.

## Material and Methods

### Selection of sampling sites and data recording

Soil samples from different regions of Switzerland and subjected to different agricultural practices were collected. Five types of crops were selected: viticulture, field crops, fruit production, vegetable crops produced in greenhouses, or vegetable crops in open fields. Fourteen Swiss states were selected for sampling since their agricultural practices included almost all the above-mentioned categories (Geneva, Vaud, Neuchâtel, Jura, Wallis, Bern, Friburg, Argovia, Zurich, Schaffhouse, Grisons, St-Gall, Thurgovia, and Ticino). For each type of crop, fields treated or not treated with azole fungicides within at least 3 years prior to sampling were chosen. This categorization was performed based on the completion of questionnaires filled out by the owners of the sampled fields. Other soil types were added to the collection including soils from each selected canton that were not known to have been subjected to any agricultural practice (natural sites) as well others from urban parks (human activity but no agricultural practice). The total number of processed samples (n = 327) and their distribution among the different cantons of the Swiss territory are given in Table 1.

**Table 1.** Distribution of sampling across Switzerland.

	Fruit production		Vegetable crops (open field)		Vegetable crops (greenhouse)		Field crops		Viticulture				
Cantons	Azoles	No azoles	Azoles	No azoles	Azoles	No azoles	Azoles	No azoles	Azoles	No azoles	Natural sites	Urban parks	Total
**Argovia**	0	0	2	0	0	0	2	4	0	2	5	5	20
**Bern**	3	3	0	0	0	0	5	6	0	0	5	5	27
**Friburg**	4	4	0	0	0	0	5	4	2	2	5	5	31
**Geneva**	1	0	0	4	2	0	0	0	0	5	5	5	22
**Grisons**	0	2	0	0	0	0	0	2	6	5	5	0	20
**Jura**	3	1	4	3	3	1	4	5	1	2	5	0	32
**Neuchâtel**	0	0	2	0	0	0	5	5	6	4	5	5	32
**Schaffhouse**	0	0	0	0	0	0	2	1	2	0	5	0	10
**St-Gall**	1	2	0	0	0	0	0	0	3	0	5	5	16
**Ticino**	2	1	2	1	1	1	0	0	3	4	5	5	25
**Thurgovia**	2	3	0	0	0	0	0	0	0	0	5	0	10
**Wallis**	4	5	5	1	2	2	2	2	4	3	5	0	35
**Vaud**	4	5	2	3	1	1	5	5	5	5	5	5	46
**Zurich**	0	0	0	0	0	0	0	1	0	0	0	0	1
**Total**	**24**	**26**	**17**	**12**	**9**	**5**	**30**	**35**	**32**	**32**	**65**	**40**	**327**

### Isolation of fungal isolates from soil samples

Selected fields were divided by drawing two diagonals, along which between six and ten soil samples were taken. Soil samples from a given site were extracted with an auger at a depth of about 20 cm, samples were mixed and an aliquot of the mix was conserved at 4°C. The auger was washed with ethanol on extraction sites after each individual soil sampling. In the laboratory, aliquots of two g were mixed with a Tween-80 solution (0,1% in distilled water) and vortexed for 10 s. After a ten min sedimentation, 100 µl of the suspension were plated onto Sabouraud (2%, Becton, Dickinson Difco TM) agar plates (SabITZ: Sabouraud itraconazole) containing chloramphenicol (Sigma-Aldrich; 0.5 g/l) and itraconazole (Sigma-Aldrich; 4 mg/l). Plates were sealed with Parafilm and incubated for 72 h at a temperature of 45°C. A rapid tape-mount staining with a lactofuchsin solution (0,1% fuchsin in 90% lactic acid) was used to identify spores of fungal isolates. Isolated fungal colonies were analyzed with Microflex LT MALDI-TOF (Matrix-assisted laser desorption/ionization-time of flight) MS (mass spectrometry) (Bruker Daltonics) and identified as *A. fumigatus* complex with the software Biotyper 3.0 using the Filamentous Fungi Library 1.0 (Bruker Daltonics). Colonies growing on SabITZ were next screened for resistance using the VIPcheck test, which consists of a 4-wells plate containing agar supplemented with three azole drugs (voriconazole at two µg/ml, itraconazole at four µg/ml, and posaconazole at 0.5 µg/ml) and a growth control. The plates were incubated at 37°C for 72 h. Assessment of azole resistance was carried out as described.^25^*Aspergillus fumigatus* isolates growing on SabITZ and in the VIPcheck test were tested for their susceptibility profiles. The *A. fumigatus* isolates were tested for *in vitro* susceptibility using the commercial and standardized broth microdilution Sensititre™ YeastOne ITAMYUCC, a modified panel YO10 with isavuconazole instead of 5-fluorocytosine (Thermo Fisher Diagnostics). Sensititre™ YeastOne is a standardized method, which corresponds to procedures of the Clinical and Laboratory Standards Institute (CLSI) and has demonstrated excellent correlation with CLSI.^26^ This is the method used in clinical routine in the reference laboratory for this study. MIC_90_ was defined as the concentration inhibiting 90% of selected isolates. Epidemiological cut-off values (ECVs), as defined by the CLSI, were used to distinguish wild-type (WT) and non-wild-type (NWT) isolates.^27^

### Sequencing

Isolates with a phenotypic resistance pattern were sequenced for the *cyp51A* gene. *Aspergillus fumigatus* DNA was extracted using a one-step procedure as described previously.^23^ The amplification of *cyp51A* was realized by polymerase chain reaction (PCR) using two cyp51A primers (5′-GAGCCGAATGAAAGTTGCCTAATTACTA-3′ and 5′-CCACAGTTTAGATAGGCTAGAAGGAG-3′). PCR products were next sequenced using two sets of primers: CYP51A-A7 (5′-TCATATGTTGCTCAGCGG-3′) and CYP51A-A5 (5′-TCTCTGCACGCAAAGAAGAAC-3′) for TR detection and Cyp51a_sq3 (5′-CATGTGCGCAATCTCTTTATC-3′ and Cyp51a_q4 (5′-CGGAAGATAGGGACTTGACGT-3′) for single sites mutation detection.

Sequencing of *tubA, cytB* and *sdhB* was carried out after PCR amplification of gene segments with primer pairs tubA-873F (5′-AGAGGGGTTAGAGAGCTTAAGG-3′) and tubA-1149R (5′-GGGTACTCTTCTGATCTCCAA-3′); cytB-802R (5′-GTGGAGTTTGCATTGGATTAGC-3′) and cytB-55F (5′-GATTCACCACAACCTGCTAATA-3′); and sdhB_188_F (5′-AATCGAGGGGAAATGTAATGCA-3)’ and sdhB_633_R (5′-CTGCGAATAGGGTCTTGAGAAC-3′).

The sequences were analysed using Geneious (Biomatters, New Zealand). Sequencing was performed by Microsynth AG (Balgach, Switzerland).

### Azole analysis

Soil extraction was based on the Accelerated Solvent extraction (ASE) method using a six-station Büchi Speedextractor E-916. The concentration of the solvent extracts was achieved by evaporation in a rotary evaporator (Büchi Rotavapor R-134). Filtration of the final soil extracts was performed using a 0.20 μm PTFE (polytetrafluoroethylene) membrane filter (Pall corporation). Purified soil extracts were collected in 2 ml PTFE screw-capped amber glass vials. Azole analysis was performed by LC-MS/MS (liquid chromatography-tandem mass spectrometry) using an Agilent Series 1200 binary pump coupled to an Agilent 6410 Triple-Quad MS using the ESI (electro spray ionisation) source. Chromatographic separation was performed using a fast-resolution C18 Zorbax Eclipse column (50 mm × 4.6 mm, 1.8 μm). The mobile phase composition included solvents A and B, which were acetonitrile and H_2_O containing 0,1% HCOOH, respectively. The mobile phase ratios were: 20% B for 2 min, then 60% B for 5 min, then 20% B for 10 min. The ESI source conditions were: nebulizing gas 3.5 bar, capillary voltage 5000 V, gas temperature 325°C, and gas flow 12 l/min. The gas used was N2. For data acquisition and processing, Agilent Mass Hunter software was used. The slopes of the calibration curves of the matrix-matched standards were compared to those obtained from the solvent-based standards, and the matrix/solvent slope ratios were calculated for each pesticide. The compounds that were analyzed were cyproconazole, difenoconazole, epoxiconazole, fenbuconazole, imazalil, mefentrifluconazole, metconazole, myclobutanil, penconazole, prochloraz, propiconazole, prothioconazole, tebuconazole, triadimenol, and triticonazole.

## Results

### Resistance profiles of recovered *Aspergillus Fumigatus* isolates

After collection of the 327 samples and positive selection of isolates on SabITZ agar plates, a total of 135 fungal isolates were recovered (Supplementary File 1). MALDI-TOF analysis identified 21 of the isolates as *Penicillium* spp., one isolate as *Neoscytalidium* spp. and 113 as *A. fumigatus*. Since the isolate selection on SabITZ is not always associated with stable azole resistance, the 113 *A. fumigatus* isolates were retested for azole resistance using the VIPcheck system. Among them, 21 were consistently growing on the tested azoles (voriconazole, posaconazole, and itraconazole). These isolates were identified as *A. fumigatus* sensu stricto by MALDI-TOF according to the mass spectrometry identification (MSI) platform, which is considered to be appropriate for the distinction between *A. fumigatus* sensu stricto and cryptic species of the section Fumigati.^28^ Separate susceptibility testing with Sensititre YeastOne was undertaken to confirm VIPcheck results. Our results showed that 21 of the initial 113 isolates that were positive by VIPcheck analysis were also azole-resistant according to the Sensititre YeastOne test and their high MICs (minimal inhibitory concentrations) (Table 2) exceeding established ECV.^27^ The remaining isolates exhibited low azole MICs typical for susceptible isolates (isavuconazole: 0.25–1 µg/ml, MIC_90_: 0.5 µg/ml; posaconazole: 0.015–0.12 µg/ml, MIC_90_: 0.06 µg/ml; voriconazole: 0.25–1 µg/ml, MIC_90_: 0.5 µg/ml; and itraconazole: 0.03–0.25 µg/ml, MIC_90_: 0.12 µg/ml; Supplementary File 1; typical susceptible sample 103 A-1 in Table 2). Azole-resistant isolates fell into two MIC profiles. One group (n = 17) showed pan-azole MIC profile (isavuconazole: 2–4 µg/ml; posaconazole: 0.25–0.5 µg/ml; voriconazole: 2–8 µg/ml; itraconazole: 16→16 µg/ml) while the other (n = 4) showed high azole MICs except for itraconazole with variable MICs (isavuconazole: >8 µg/ml; posaconazole: 0.25–0.5 µg/ml; voriconazole: >8 µg/ml; itraconazole: 0.25–16 µg/ml). Sequencing of *cyp51A* in these isolates revealed that these two groups contained mutations associated with resistance. Pan-azole resistance was associated with a L98H mutation coupled with a TR34 in the *cyp51A* promoter, while the other resistance profile was linked to Y121F/T289A mutations with a 46-bp tandem repeat (TR46) (Table 2). Taken from these data, azole resistance (taking voriconazole MICs data) was detected in 5.8% of all sampled soils and in 18.6% of all recovered *A. fumigatus* isolates.

**Table 2.** Characteristics of azole-resistant *Aspergillus fumigatus* isolates.

General informations				MIC (µg/ml)								Genotype			
Isolate^1^	Canton	Soil type	Treatment	AND	MF	CAS	ISA	POS	VRC	ITZ	AmB	*cyp51A*	*t u bA*	*cytB*	*sdhB*
11 A	Vaud	Field crops	Yes	0.015	0.008	0.008	>8	0.25	>8	0.25	1	TR46/Y121F/T289A	wt	wt	wt
18 B	Vaud	Field crops	No	0.015	0.008	0.008	2	0.25	2	>16	0.5	TR34/L98H	wt	wt	wt
25 A	Vaud	Viticulture	Yes	<0.015	<0.008	<0.008	>8	0.25	>8	0.25	2	TR46/Y121F/T289A	F200Y	G143A	H270Y
47 B-1	Jura	Fruit production	No	<0.015	<0.008	<0.008	2	0.25	2	>16	1	TR34/L98H	wt	wt	wt
6 A	Vaud	Fruit production	No	0.015	0.008	0.008	4	0.25	2	>16	1	TR34/L98H	wt	wt	wt
87 A	Neuchâtel	Vegetable crops	No	<0.015	<0.008	<0.008	2	0.25	2	>16	2	TR34/L98H	F200Y	wt	wt
332 A1	Neuchâtel	Urban park	—	<0.015	<0.008	<0.008	4	0.25	4	>16	1	TR34/L98H	wt	wt	wt
185 B	Wallis	Field crops	Yes	0.015	0.008	0.008	>8	0.5	>8	>16	1	TR46/Y121F/T289A	F200Y	G143A	wt
151 B	Wallis	Field crops	Yes	0.015	0.008	0.008	>8	0.5	>8	>16	1	TR46/Y121F/T289A	F200Y	wt	wt
346 B1	Geneva	Urban park	—	0.015	0.008	0.015	4	0.5	4	>16	1	TR34/L98H	wt	wt	wt
245	Ticino	Vegetable crops	Yes	0.015	0.008	0.008	4	0.25	4	16	0.25	TR34/L98H	wt	wt	wt
223	Shaffhouse	Natural site	No	0.015	0.008	0.008	4	0.5	4	16	0.25	TR34/L98H	wt	wt	wt
158 a2	Wallis	Viticulture	Yes	0.015	0.008	0.008	4	0.5	4	16	0.25	TR34/L98H	wt	wt	wt
221	Shaffhouse	Natural site	No	0.015	0.008	0.008	4	0.5	2	16	1	TR34/L98H	wt	wt	wt
305	Bern	Field crops	No	0.015	0.008	0.008	4	0.25	4	16	1	TR34/L98H	wt	wt	wt
218	Shaffhouse	Viticulture	No	0.015	0.008	0.008	4	0.25	4	16	1	TR34/L98H	wt	wt	wt
153	Wallis	Viticulture	No	0.015	0.008	0.008	4	0.25	4	16	1	TR34/L98H	wt	wt	wt
260	Thurgovia	Fruit production	No	0.015	0.008	0.008	4	0.5	4	>16	1	TR34/L98H	wt	wt	wt
258-1	Thurgovia	Fruit production	No	0.015	0.008	0.008	4	0.25	4	>16	0.5	TR34/L98H	wt	wt	wt
103-A1	Neuchâtel	Natural site	No	<0.015	<0.008	<0.008	0.5	0.03	0.25	0.06	0.5	wt	wt	wt	wt

Abbreviations: AND, Anidulafungin; MF, Micafungin; CAS, Caspofungin; ISA, isavuconazole; POS, posaconazole; VRC, voriconazole; ITZ, itraconazole; AmB, amphotericin B; wt, wild type; and —, no treatment.

1 Soil samples 6 and 185 contained *A. fumigatus* duplicates, only one of which is listed (6 A and 185 B).

**Figure 1.**
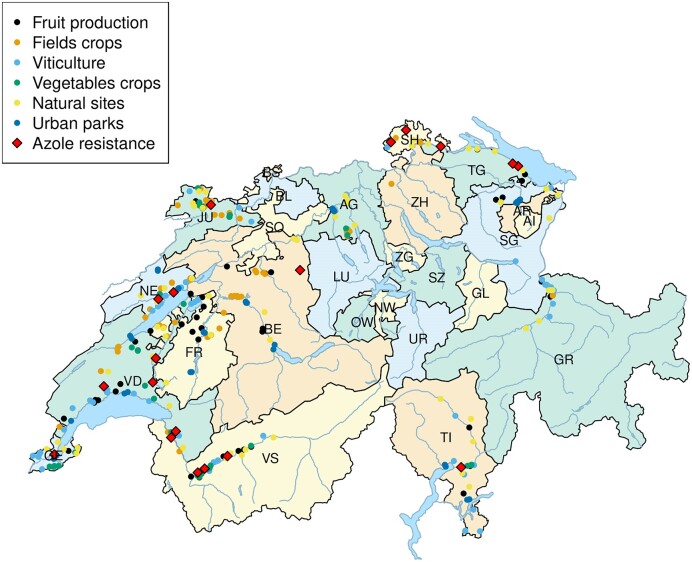
Distribution of samples and azole-resistant isolates in Switzerland. Cantons are designated by abbreviations. The sampled sites contained 108 and 114 azole-treated and azole-untreated sites, respectively. The number of natural sites and urban parks was 65 and 40, respectively. Azole resistance was detected in 19 different sites. Sites were mapped by their GPS (global positioning system) coordinates. The digital template map was imported from the Federal Office of Statistics (Bern) and the map was generated with the R package bfsMaps.

**Figure 2.**
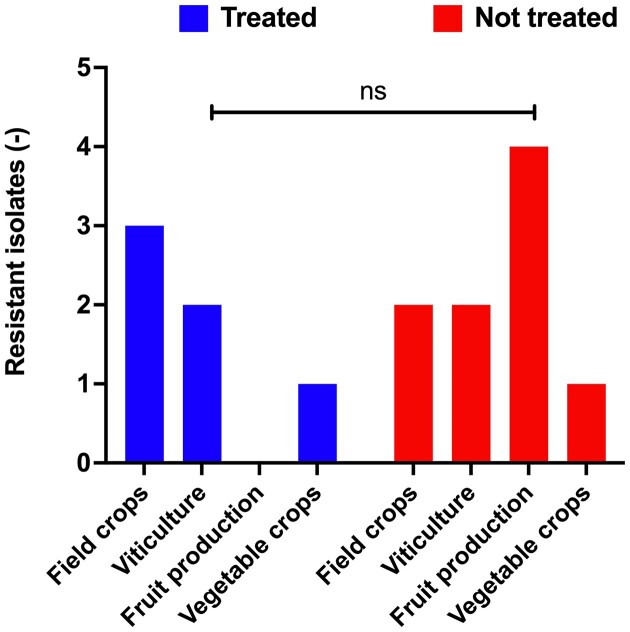
Distribution of azole-resistant isolates among different soil types. Natural sites and urban parks were not included since azole exposure cannot be formally excluded. Two-way Anova tests were performed using Graph Prism 9.5.1; ns: not significant.

Figure 1 shows the repartition of soil samples over the Swiss country and the distribution of azole-resistant isolates (red diamonds). Azole-resistant isolates are scattered in the entire country, and they were present in all types of agricultural lands and even in natural sites (Table 2). All four isolates carrying the TR46/Y121F/T289A mutation were isolated from agricultural lands where azole fungicides had been used in recent years. Other than that, no significant association could be made between the occurrence of azole resistance and recorded fungicide treatments (Figure 2). In addition, two different soils originating from natural sites free of agricultural activity contained azole-resistant isolates (223 and 158 a2; TR34/L98H). These data suggest that azole resistance is not necessarily obtained by a selective pressure resulting from the presence of agricultural fungicides in sampled soils. The ability of *A. fumigatus* to produce a high number of airborne conidia that spread over large territories, and among them some carrying azole resistance genes, may explain this observation.

### Occurrence of additional fungicide resistance genes in *A. Fumigatus*

The obtained *A. fumigatus* collection was also tested for other resistance genes that are known to emerge when specific substances are used in agricultural practice. These substances include the classes of benzimidazoles, strobilurins and succinate dehydrogenase inhibitors (SDHI). Mutations in *tubA* (resistance to benzimidazoles), *cytB* (resistance to quinone outside inhibitors [QoI's] strobilurins), and *sdhB* (resistance to SDHI) genes are known to confer resistance to the substances in question.^29^ The 19 azole-resistant isolates as well as a same number of susceptible strains were systematically tested for these mutations (Supplementary File 1). Sequencing analysis revealed that all the azole-susceptible isolates did not show any mutation in the *tubA, cytB*, and *sdhB* genes. Most of the azole-resistant strains did not show mutations in these genes either. Only four distinct isolates contained mutations conferring resistance to non-azole fungicides, all of them from agricultural lands and three of them with known azole fungicide applications (Table 2). These mutations correspond to those published earlier and causing resistance to the above-mentioned fungicides. One specific isolate (25 A) even exhibited mutations in all the three tested genes. Interestingly the detected fungicide mutations were all associated with the occurrence of the azole resistance profile TR46/Y121F/T289A. Taken together, these results strongly suggest that *A. fumigatus* has been exposed at some point to several types of fungicides used in the agriculture. *Aspergillus fumigatus* consequently adapts by the acquisition of mutations conferring drug resistance.

### Detection of azoles in sampled soils

**Figure 3.**
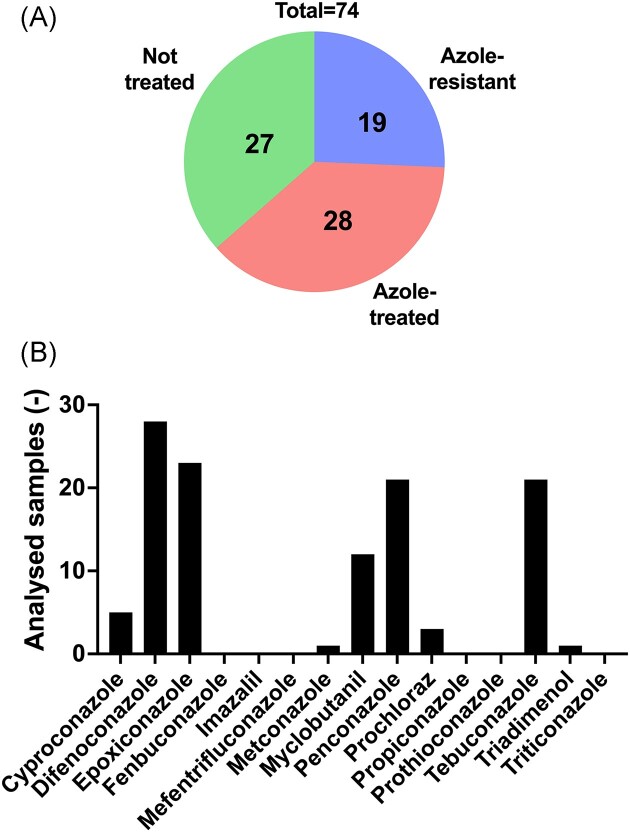
Azole analysis in soil samples. Panel A: Distribution of samples subjected to azole detection analysis; Panel B: Occurrence of azoles in the entire panel of analyzed samples (n = 74). The detection limit of azoles by the method was <0.1 μg/kg soil (except for propiconazole and prothioconazole, the limit of the method was at <0.5 µg/kg soil).

**Figure 4.**
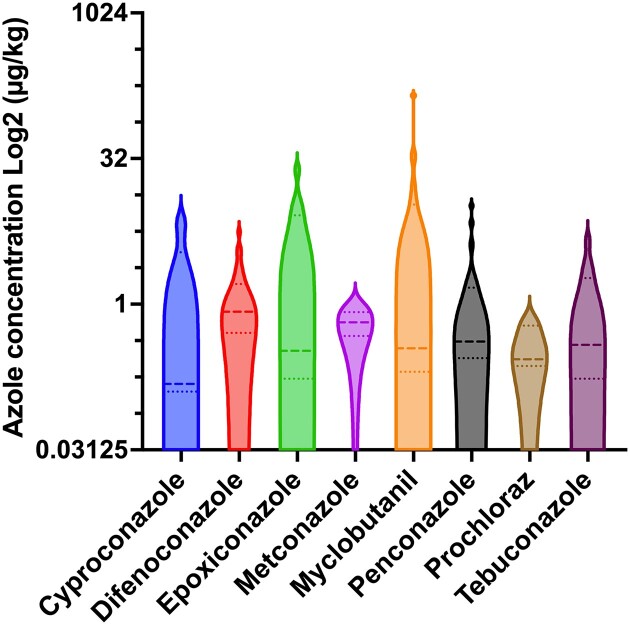
Concentration ranges of azoles detected in soils. Propiconazole and triadimenol plots are not shown since only one value was obtained. Quartiles and median values are illustrated by dotted and dashed lines, respectively.

**Figure 5.**
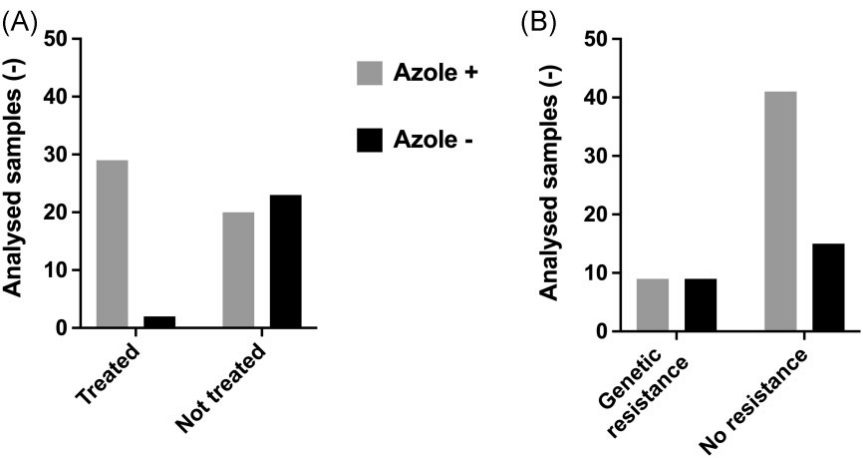
Azole detection in soil samples. Panel A: Azole detection in relationship with obtained treatment declarations. Panel B: Distribution of genetic resistance in relationship with azole detection. Azole +: azoles detected in soil samples and Azole -: absence of azoles.

Given that azole resistance was occurring in specific soil samples, one hypothesis is that the presence of azoles in soils could have favored the development of resistance. We therefore carried out a chemical analysis of soils (n = 74) by LC-MS/MS containing resistant or susceptible *A. fumigatus* isolates. The distribution of the samples according to the treatments and their origin is visible in Figure 3A. Figure 3B shows that different azoles used currently in agriculture were detectable. The detection limit of the method was at <0.1 µg/kg soil (except for propiconazole and prothioconazole, the limit of the method was at <0.5 µg/kg soil). Contemporary azoles (commercialized after 1985) such as difeconazole, epoxiconazole, penconazole, and tebuconazole are mostly detected in the samples (Figure 3B). While 24 samples were negative for azole detection, 15, ten, 15, and nine samples were positive for one, two, three, and four azoles, respectively (Supplementary File 2). Azoles were most frequently detected in soils from field crops, fruit production, and viticulture. The obtained quantitative data showed high variations in azole concentrations (Figure 4) but in general, the median concentration varied between 0.25 and 0.55 µg/kg soil. If we consider the declarations of treatments obtained by producers, we observe that most samples from treated soils contained azoles. On the other hand, approximately 50% of the soils that were declared as untreated contained traces of azoles (Figure 5A). Taking into account that treatment declarations were accurate, this suggests that azoles have fairly long half-lives, given that the declaration threshold for untreated soil was 3 years. This is supported by the fact that, among the detected azoles in this work, cyproconazole, epoxiconazole, metconazole, myclobutanil, and prochloraz have median half-lives over a threshold of 500 days (Supplementary Figure 1).

Figure 5B shows that genetic azole resistance is only associated with the detection of azoles in 50% of cases. However, it cannot be excluded that azoles had been previously applied in those fields, inducing the resistance of the isolates recovered in this study. Nonetheless, azole-resistant isolates were found in natural sites that are not known to have been subjected to any agricultural practice. Altogether, these results suggest that the presence of azole-resistant *A. fumigatus* isolates is not necessarily linked to the exposure to azoles in soils.

## Discussion

Azole resistance in *Aspergillus fumigatus* is an increasing global health concern and is now observed at the worldwide scale.^30^ At least two routes of azole resistance acquisition have been described: on one hand a long-term azole therapy in clinical settings, and on the other hand the use of azole fungicides in the agriculture. Environmental triazole resistance in *A. fumigatus* is characterized by genetic signatures involving the azole target Cyp51A. Principally, the genetic changes consist in TRs in the promoter region of *cyp51A* resulting in increased *cyp51A* expression and are associated with coding region point mutations that alter azole affinity to the target. The most commonly occurring resistance alleles typical for environmental azole resistance acquisition, TR34/L98H and TR46/Y121F/T289A, are associated with azoles used in medicine and in the agriculture.^31^ Interestingly, a recent study performed in the UK, in which 218 *A. fumigatus* isolates were sampled from the environment and the clinic, showed that azole-resistant isolates were mostly clustered in a specific sub-population group (Clade A).^15^ This population structure is in agreement with other studies.^15,32,33^ These data suggest that the azole resistance based on TRs in *cyp51A* stemmed from a common ancestor and is restricted to genetically related isolates. In this study, we addressed for the first time the occurrence of *A. fumigatus* azole resistance in soils of a large part of the Swiss territory. Unsurprisingly, azole resistance was detected and genetic analysis revealed the presence of two signatures typical for environmental resistance acquisition, i.e., TR34/L98H and TR46/Y121F/T289A. Up to now, the TR46/Y121F/T289A genetic profile has not been reported in Switzerland. These two profiles were associated with specific MIC profiles in *A. fumigatus*, in which the TR46/Y121F/T289A mutations elevated isavuconazole and voriconazole MICS, as observed by others.^34,35^ Whether or not azole-resistant isolates could be associated with a specific population structure as above-mentioned remains to be established.

Interestingly, while the current study was on-going, a clinical prospective study for azole-resistant *A. fumigatus* isolates was carried out in different Swiss hospitals. Among the recovered azole-resistant isolates from patients (n = 4), three exhibited the TR34/L98H mutation and one a single change in *cyp51A* (M220K).^24^ The TR34/L98H mutation was found in patients with leukemia and IA, and in two non-immunocompromised patients (chronic pulmonary obstructive disease and cystic fibrosis) with lung colonization. In two cases, azole resistance occurred prior to azole treatment. These data again support the environmental origin of azole resistance.

We attempted to associate azole resistance with specific agricultural practices (crop type, use of azole fungicides), but no clear trend could be deduced from our analysis. No impact of selection pressure exerted by spraying azole fungicides could be observed on the *A. fumigatus* Swiss population. The effect of selection may have been masked by the strong gene flow to which this population is subjected due to the production of numerous conidia. Nonetheless, it is worth noting that the four isolates with the TR46/Y121F/T289A mutation were recovered from agricultural fields with known azole treatments. This observation suggests that this mutation might be associated with azole fungicide application in agricultural lands. Indeed, tebuconazole (an azole used in agriculture) has been shown to induce resistance in *A. fumigatus* associated with the TR46/Y121F/T289A mutation.^36^ Furthermore, the TR46/Y121F/T289A mutation in clinical cases has been shown to be closely related to those from environmental origin, suggesting an exclusively environmental origin of this mutation.^14^

Other studies have reported that the presence of azoles in specific environments or materials (compost sites, flower bulb waste, wood chippings, sawmills) may have facilitated the emergence of azole-resistant isolates.^34,35,37–39^ In a specific study in which soils were sampled for both azole resistance screening and azole detection, authors reported azole concentrations in a range similar to those reported here (1–300 µg/kg). Like our observations, the presence of azole-resistant isolates was not necessarily linked to the detection of azoles in soil samples.^34^ Several explanations could be provided to understand this observation. First, azoles can be degraded in soils. Their half-lives range from days to years, however, these values are highly dependent on the soil types and temperature variations.^31^ Moreover, soils have different water permeabilities and drainage efficiencies and azoles may only shortly be in contact with *A. fumigatus*.^34^ Secondly, *A. fumigatus* is conidia-proficient and azole-resistant conidia may be dispersed over large territories. Collecting azole-resistant isolates in soils without azole-pre-exposure is therefore possible. The best illustration of this hypothesis is the isolation of azole-resistant isolates in soils devoid of agricultural activities (natural sites).

The fungicide-driven azole resistance hypothesis is supported by the acquisition of additional fungicide resistance mutations. Here we showed that several azole-resistant isolates carried mutations in genes (*tubA, cytB*, and *sdhB*) that are responsible for resistance to specific fungicides (benzimidazoles and SDHI). These findings corroborate with other studies in which *A. fumigatus* isolates were screened for these mutations. Notably, three out of the four isolates carrying the TR46/Y121F/T289A mutation possessed a mutation in at least one of these genes (*tubA*) (Table 2). These results further suggest the environmental acquisition of the TR46/Y121F/T289A mutation, as previously suggested.^14^ In a milestone study, Fraaije et al.^40^ addressed fungicides MICs of several azole-resistant *A. fumigatus* isolates and revealed high MICs to the methyl benzimidazole carbamate (MBC) fungicide carbendazim, to the QoI fungicide pyraclostrobin, and to the SDHI fungicide boscalid. The authors identified mutations resulting in amino acid substitutions in *tubA* (F200Y), in *cytB* (G143A), and in *sdhB* (H270Y and H270R) responsible for resistance to the non-azole fungicides. These findings were later replicated by separate studies.^29,41^ Multi-resistance to non-azole fungicides by detection of mutations in the three genes *tubA, cytB*, and *sdhB* was observed here for one isolate but was also reported in these studies.^29,41^ These data strongly support the hypothesis of fungicide resistance acquired environmentally. Whether these drug resistance mutations were acquired sequentially or whether genetic exchange within *A. fumigatus* isolates allowed the accumulation of drug-resistance genes is currently not possible to answer.

In conclusion, this study further illustrates the occurrence of azole resistance in *A. fumigatus* in soils during a specific period (2019–2021). It is now widely recognized that agricultural fungicides have been drivers for a selective pressure for resistance in *A. fumigatus*.^42^ While we focused on this specific fungal species since it is coupled to human health, other fungal species may be also affected. Reducing the use of agricultural fungicides in the environment may impact on the occurrence of resistance in fungi. It is, therefore, necessary to support in the future continuing broad surveillance of resistance to accurately report trends in the resistant fungal population.

## Supplementary Material

myad110_Supplemental_Files
