# Supplementary figures and images for: Occurrence of Aspergillus fumigatus azole resistance in soils from Switzerland
Source: Med Mycol. 2023 Nov 1;61(11):myad110. doi: 10.1093/mmy/myad110 (PMC10653585; doi:10.1093/mmy/myad110)

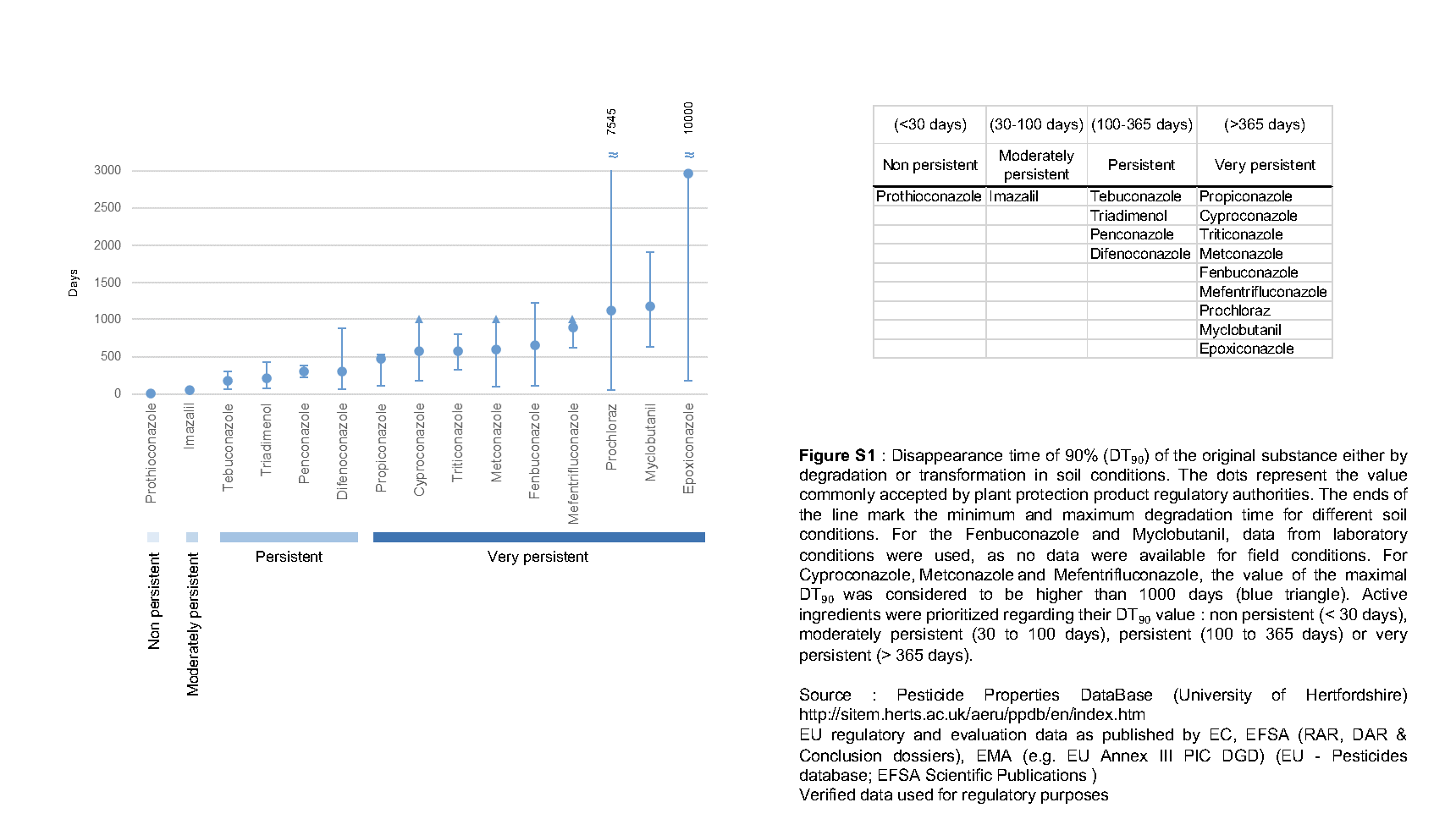

Supplement: myad110_Supplemental_Files [file myad110_supplemental_files.zip › mm-2023-0213-File009.tiff]
